# Measuring neuropsychiatric symptoms in early dementia patients using speech analysis

**DOI:** 10.1192/j.eurpsy.2022.461

**Published:** 2022-09-01

**Authors:** A. König, E. Mallick, N. Linz, R. Zegahri, V. Manera, P. Robert

**Affiliations:** 1 Institut national de recherche en informatique et en automatique (INRIA), Stars Team, Nice, France; 2 ki:elements, Ug, Saarbrücken, Germany; 3 FRIS-University Côte d’azur, Cobtek (cognition-behaviour-technology) Lab, Nice, France

**Keywords:** Neuropsychiatric symptoms, Depression, apathy, speech analysis

## Abstract

**Introduction:**

Certain neuropsychiatric symptoms (NPS), namely apathy, depression and anxiety demonstrated great value in predicting dementia progression representing eventually an opportunity window for timely diagnosis and treatment. However, sensitive and objective markers of these symptoms are still missing.

**Objectives:**

To investigate the association between automatically extracted speech features and NPS in early-stage dementia patients.

**Methods:**

Speech of 141 patients aged 65 or older with neurocognitive disorder was recorded while performing two short narrative speech tasks. Presence of NPS was assessed by the Neuropsychiatric Inventory. Paralinguistic markers relating to prosodic, formant, source, and temporal qualities of speech were automatically extracted, correlated with NPS. Machine learning experiments were carried out to validate the diagnostic power of extracted markers.

**Results:**

Different speech variables seem to be associated with specific neuropsychiatric symptoms of dementia; apathy correlates with temporal aspects, anxiety with voice quality and this was mostly consistent between male and female after correction for cognitive impairment. Machine learning regressors are able to extract information from speech features and perform above baseline in predicting anxiety, apathy and depression scores.

**Conclusions:**

Different NPS seem to be characterized by distinct speech features which in turn were easily extractable automatically from short vocal tasks. These findings support the use of speech analysis for detecting subtypes of NPS. This could have great implications for future clinical trials.

**Disclosure:**

No significant relationships.

